# Major depressive disorders increase the susceptibility to self-reported infections in two German cohort studies

**DOI:** 10.1007/s00127-022-02328-5

**Published:** 2022-07-05

**Authors:** Henning Elpers, Henning Teismann, Jürgen Wellmann, Klaus Berger, André Karch, Nicole Rübsamen

**Affiliations:** Institute of Epidemiology and Social Medicine, University of Münster, Albert-Schweitzer-Campus 1, 48149 Münster, Germany

**Keywords:** Major depressive disorder, Infections, Disease susceptibility, Causal inference

## Abstract

**Introduction:**

In several claims-based studies, major depressive disorder (MDD) has been associated with increased risk of hospitalization due to acute infections. It remains unclear if this is a causal effect, and if it generalizes to an increased susceptibility to infections.

**Methods:**

We used data of the BiDirect (*n* = 925) and the HaBIDS (*n* = 1007) cohort studies to estimate the effect of MDD on self-reported infections, which were assessed with identical infection susceptibility questionnaires in both studies. We used the Center for Epidemiologic Studies Depression Scale (CES-D) to examine if there was a dose–response relationship between depressive symptom severity and self-reported infections.

**Results:**

BiDirect participants with MDD diagnosis (48%) had a higher risk of lower respiratory tract infections (incidence rate ratio 1.32, 95% confidence interval [1.00–1.75]), gastrointestinal infections (1.68 [1.30–2.16]) and fever (1.48 [1.11–1.98]) after adjusting for confounders identified by a directed acyclic graph approach. There was a dose–response relationship, i.e. individuals with higher CES-D scores reported more infections. Effect sizes were similar in HaBIDS (4% individuals with MDD).

**Conclusion:**

We found increased risks of mild infections in patients with MDD diagnosis and a dose–response relationship between depressive symptom severity and infection frequency. While causal immunological pathways remain unclear, the results of our study might contribute to a change in prevention strategies, e.g. by recommending vaccination against influenza and *S. pneumoniae* to MDD patients because observed effect sizes in our study are similar to those of patients with cardiovascular and metabolic diseases for which the respective vaccinations are recommended.

**Supplementary Information:**

The online version contains supplementary material available at 10.1007/s00127-022-02328-5.

## Introduction

Major depressive disorders (MDDs) have a profound and pervasive impact on physical health, for example, by causing immuno-inflammatory dysregulation [1]. Consequently, a link between MDD and susceptibility to infections has been proposed [2]. A register-based study from Denmark revealed MDD to be a risk factor for severe infections (e.g. hepatitis infection, lower respiratory tract infection, sepsis) leading to contact with the healthcare system [3]. In a systematic review, a variety of psychosocial factors (including the diagnosis of MDD) were found to be associated with acute respiratory tract infections (RTI) [4]. These findings are supported by more specific findings for different types of RTI. In patients with pneumonia, for example, MDD has been identified as a risk factor for hospitalization [5], mortality [6, 7], admission to an intensive care unit and the need for mechanical ventilation [7]. Similar analyses have been performed for other infectious diseases, such as gastrointestinal infections, which have as well been reported to be associated with MDD [8].

Previous studies have had specific limitations due to the use of data from health registers, hospitals or health insurances in most studies, leading to (i) the focus on more severe infectious diseases, (ii) missing information about MDD besides the ICD diagnosis (e.g. severity, medication intake) as well as (iii) a lack of a sufficiently large group of individuals with MDD to analyse different subgroups. The present study sought to investigate the effect of MDD on the occurrence of infections in a population-based setting. Specifically, this study aimed to investigate the incidence of mild infections, which do not necessarily lead to contact with the healthcare system, based on self-reports of depressed and non-depressed individuals; to investigate whether there is a dose–response relationship between the severity of depressive symptoms and risk of infections and to estimate incidence rate ratios by adjusting for confounders identified via a directed acyclic graph (DAG) approach.

## Materials and methods

### Study design and population

The BiDirect Study [9, 10] is an observational, prospective cohort study originally designed to investigate the bidirectional relationship between depression and (subclinical) arteriosclerosis. Between 2010 and 2013, BiDirect enrolled participants aged 35 to 66 years into three distinct cohorts: (1) patients hospitalized due to an acute episode of depression at the time of recruitment (*N* = 999), (2) patients shortly after myocardial infarction or an acute coronary event at the time of recruitment (*N* = 347) and (3) population-based control subjects randomly invited from the registry of the city of Münster, Germany (*N* = 912). The thorough assessment of depressive symptoms is a key element of BiDirect: For patients from cohort 1, assessment of depression was conducted during recruitment. For participants from cohorts 2 and 3, diagnosis of depression took place during the baseline examination at the study centre [10]. BiDirect participants underwent up to three follow-up examinations, with an average of two to three years between examinations. The third follow-up examination between 2018 and 2020, which included a questionnaire on the infection data analysed here, was completed by 1024 participants from the BiDirect cohorts 1 and 3 (participants from the myocardial infarction cohort were excluded from our analyses). In our analysis, we restricted all analyses to this population and then excluded all participants with self-reported intake of systemic immune-modulating medication (*n* = 32) based on ATC codes (i.e. corticosteroids for systemic use, antineoplastic agents, immunostimulants, immunosuppressants).

The HaBIDS study (Hygiene and Behaviour Infectious Diseases Survey) was a longitudinal online panel aiming to assess hygiene practices and behaviour regarding various infectious diseases in the federal state of Lower Saxony, Germany. The detailed description of the applied methodology is presented elsewhere [11, 12]. In brief, almost 27,000 males and females between 15 and 69 years of age were invited to participate in the panel. Potential participants were randomly selected from the population registries in urban (Braunschweig, Salzgitter and Wolfenbüttel) and rural areas (Vechta). Each month, participants completed questionnaires on different aspects of infectious diseases. Among 2379 individuals who had consented to participate in the panel (8.9% initial response rate) 1151 filled in both a questionnaire about medical history (in summer 2015) and a questionnaire about frequency of infections and infection-associated symptoms within the preceding 12 months (in spring 2014). In addition, HaBIDS participants were asked in May 2015 (873/1151) and in May 2016 (788/1151) about the frequency of RTI (e.g. cold, flu or otitis media) within the preceding 12 months, respectively. We refer to these variables as RTI from the seasons 2014/15 and 2015/16.

### Definition of exposure

The primary exposure of interest was life-time diagnosis of MDD at baseline. In BiDirect, all participants in cohort 1 were exposed (*N* = 361) as well as participants in cohort 3 who had received an MDD diagnosis at baseline (*N* = 79). In HaBIDS, exposure classification was determined by the self-report of MDD in the medical history survey. To investigate the effect of recent depressive symptom severity on infectious diseases, we used in BiDirect the German version of the Center for Epidemiologic Studies Depression Scale (CES-D [13]) as well as treatment with antidepressant medication. The CES-D summary score was categorized in four different categories as described in the literature [14]: not depressed (< 10 points), mild depression (10–15 points), moderate depression (16–24 points) and severe depression (≥ 25 points). Classification of the CES-D score was independent of the life-time diagnosis of MDD, i.e. a participant without life-time diagnosis of MDD could be categorized as “depressed” if the recent CES-D score was above 9 points. Treatment with antidepressant medication was coded by ATC codes (i.e. non-selective monoamine reuptake inhibitors, selective serotonin reuptake inhibitors, non-selective monoamine oxidase inhibitors, monoamine oxidase A inhibitors, lithium, other antidepressants).

### Definition of outcome

The outcomes of interest were self-reported infectious diseases and infection-related variables within the 12 months preceding the respective questionnaire [15]. The BiDirect questionnaire was filled in during the third follow-up examination, and included upper respiratory tract infections (URTI), lower respiratory tract infections (LRTI), cystitis, gastrointestinal (GI) infections, fever and intake of antibiotics. From the HaBIDS study, we used information on the frequency of URTI, LRTI, RTI from the seasons 2014/15 and 2015/16, as well as on cystitis, long-lasting coughing, fever, diarrhea and herpes labialis in spring 2014. For all variables of BiDirect, except for intake of antibiotics, and for all variables of HaBIDS, except for RTI in the 2014/15 and 2015/16 seasons, the questionnaire included seven answer options. For the analysis of all answers including a range of values, we used the lower one (all used values in brackets): “Never” (0), “Once” (1), “Twice” (2), “3 to 4 times” (3), “5 to 6 times” (5), “More than 6 times” (7) and “Don’t know” (Missing). We created the variable “any RTI” as the sum of URTI and LRTI. For the intake of antibiotics in BiDirect and for RTI from 2014/15 and 2015/16 in HaBIDS, we used the following values: “Never” (0), “Once” (1), “Twice” (2), “3 to 4 times” (3), “More than 4 times” (5) and “Don’t know” (Missing). We excluded participants (BiDirect: *N* = 62; HaBIDS: *N* = 144) who had any missing value in the outcome variables (except for RTI in 2014/15 and 2015/16 because they were, by design, only completed by a subset of participants). If the outcome variable has missing values, then the incomplete cases do not contain any information about the exposure coefficient; in these cases, complete case analysis is recommended [16].

### Definition of covariables

Confounders of the effect of MDD diagnosis on self-reported infections were identified by using a DAG (Online Resource 1). Whether a variable or an arrow between two variables was included and how these arrows were directed were decided based on published study results (Online Resource 2). There was evidence for bidirectional associations between five pairs of variables. For the DAG, we decided on one direction (as reported in Online Resource 2). Using the R package *dagitty* (version 0.3–1, code available at zivgitlab.unimuenster.de/ruebsame/mdd_infection_germany_bidirect_habids) [17], we identified the following minimal sufficient adjustment set (MSAS): age, sex, socioeconomic status, body mass index (BMI), household size, smoking, alcohol intake, physical activity, nutritional status, stress, intake of proton pump inhibitors, asthma bronchiale, chronic obstructive pulmonary lung disease (COPD), diabetes mellitus, heart failure, chronic kidney disease, stroke and cancer. The MSAS did not change if the direction of the five arrows in question was reversed in the DAG. We checked whether the assumptions encoded in the DAG were consistent with the data using dagitty’s function localTests.

In the analysis of the BiDirect data, we adjusted for age, sex, socioeconomic status (years of education, marital status, income), BMI, household size, smoking, alcohol intake (g/d), physical activity (IPAQ-level [18]), stress (PSS-14 [19]), intake of proton pump inhibitors, chronic lung disease (covering asthma bronchiale and COPD), diabetes mellitus, heart failure, chronic kidney disease and stroke. We did not adjust for nutritional status because it was measured with uncertainty. We adjusted for cancer by excluding all participants with intake of systemic immune-modulating medication as described above. In HaBIDS, the dataset included the following variables to adjust for age, sex, socioeconomic status (education level, marital status, income), household size, smoking, alcohol intake (AUDIT-C [20]), stress (PSS-4 [19]) and comorbidities (i.e. chronic lung disease, diabetes mellitus, heart failure, chronic kidney disease, stroke). We were not able to adjust for BMI, intake of proton pump inhibitors, physical activity, nutritional status and cancer in the analysis of the HaBIDS study.

### Statistical analysis

All analyses were performed with R version 4.1.3 (www.R-project.org). We used negative binomial regression models to estimate incidence rate ratios (IRR) and 95% confidence intervals (CI). P value functions were calculated by using the R package *pvaluefunctions* (version 1.6.2) [21]. To evaluate differences of infection frequency in participants with vs. without antidepressant treatment, we categorized BiDirect participants into three groups: no MDD (reference group; *n* = 485), MDD without medication (*n* = 198) and MDD with medication (*n* = 242). Missing values in confounder variables were imputed (R package mice [22] version 3.14.0) with five multiple imputations. Regression coefficients were estimated in each imputed dataset separately and pooled afterwards. Missing values in outcome variables were not imputed as discussed above.

### Quantitative bias analysis

As MDD diagnosis was ascertained by trained staff in BiDirect, but based on self-report in HaBIDS, we explored whether misclassification of this exposure biased our estimate of effect in HaBIDS. We assumed that any misclassification would be non-differential, i.e. not dependent on the outcome, so we used a wide range of values for sensitivity and specificity (10–100%) to correct the observed estimate of effect with the method suggested by Fox et al. [23].

The outcome could be misclassified due to mood-congruent memory, i.e. that individuals with depression recall more negative biographical events than controls without depression [24]. Using the BiDirect data (where we could adjust for nearly all confounders), we explored three different scenarios: 1) that all participants with recent CES-D score ≥ 16 points; 2) that 50% and 3) that 90% of all participants with MDD diagnosis reported too many infections. In each scenario, we reclassified the respective participants to the adjacent lower outcome category (e.g. from “Twice” to “Once”) and re-estimated the IRR as described above. We did not reclassify participants from “Once” to “None” because we assumed that participants with MDD diagnosis would falsely report the number of infections, but not their actual occurrence. The R code for the bias analyses is available at zivgitlab.uni-muenster.de/ruebsame/mdd_infection_germany_bidirect_habids.

## Results

Of all 925 participants of the BiDirect cohort study included into our analysis, 440 participants (48%) had received a diagnosis of MDD in the baseline examination (Table 1). Forty-one of all 1007 participants of the HaBIDS study with information about medical history reported a diagnosis of depression in their lifetime at baseline (4%) (Table 1). Most assumptions encoded in the DAG were consistent with the data (Online Resource 3).

**Table 1:** Characteristics of the BiDirect (*n* = 925) and HaBIDS (*n* = 1007) study populations

Median (IQR); *n* (%)	BiDirect		HaBIDS	
	Depression		Depression	
	No *N* = 485	Yes *N* = 440	No *N* = 966	Yes *N* = 41
URTI	1 (1, 2)	1 (1, 2)	2 (1, 3)	2 (1, 3)
LRTI	0 (0, 0)	0 (0, 1)	0 (0, 0)	0 (0, 1)
Any RTI	1 (1, 2)	2 (1, 3)	2 (1, 3)	2 (1, 3)
Any RTI in season 2014/2015	–	–	1 (1, 2)	1 (0, 2)
Missing	–	–	241	15
Any RTI in season 2015/2016	–	–	1 (0, 2)	1 (1, 2)
Missing	–	–	310	8
Cystitis	0 (0, 0)	0 (0, 0)	0 (0, 0)	0 (0, 0)
GI infection	0 (0, 1)	0 (0, 1)	1 (0, 2)	1 (0, 2)
Fever	0 (0, 0)	0 (0, 1)	0 (0, 1)	0 (0, 1)
Intake of antibiotics	0 (0, 1)	0 (0, 1)	–	–
Herpes labialis	–	–	0 (0, 1)	0 (0, 1)
Cough lasting more than 4 weeks	–	–	0 (0, 0)	0 (0, 1)
Severity of depressive symptoms				
No depression	368 (76%)	135 (31%)	–	–
Mild	80 (16%)	69 (16%)	–	–
Moderate	23 (5%)	111 (25%)	–	–
Severe	14 (3%)	125 (28%)	–	–
Antidepressant medication				
No MDD	485 (100%)	0 (0%)	–	–
MDD without Medication	0 (0%)	198 (45%)	–	–
MDD with Medication	0 (0%)	242 (55%)	–	–
Age	61 (54, 67)	58 (53, 64)	46 (35, 56)	50 (34, 56)
Missing	0	1	–	–
Women	243 (50%)	270 (61%)	570 (59%)	33 (80%)
Years of education	16 (13, 18)	15 (13, 17)	–	–
Missing	0	1	–	–
Highest educational level				
Still student	–	–	21 (2%)	1 (2%)
Lower secondary education or apprenticeship	–	–	266 (28%)	14 (34%)
University entrance qualification	–	–	260 (27%)	9 (22%)
University degree	–	–	415 (43%)	17 (41%)
Missing	–	–	4	0
Marital status				
Married	315 (70%)	243 (59%)	595 (62%)	22 (54%)
Divorced	49 (11%)	81 (20%)	73 (8%)	5 (12%)
Single	64 (14%)	64 (15%)	274 (29%)	13 (32%)
Widowed	22 (5%)	26 (6%)	19 (2%)	1 (2%)
Missing	35	26	5	0
Income	3001–4000 € (2001–3000 €, 5001–6000 €)	2001–3000 € (1001–2000 €, 4001–5000 €)	3000–3999 € (2250–2999 €, 4000–4999 €)	2250–2999 € (1750–2249 €, 4000–4999 €)
Missing	53	39	–	–
BMI	26 (24, 29)	28 (25, 32)	–	–
Missing	0	2	–	–
Household size				
1 person	89 (20%)	128 (31%)	133 (15%)	7 (18%)
2 persons	268 (60%)	205 (49%)	304 (35%)	17 (45%)
3 persons	46 (10%)	49 (12%)	210 (24%)	10 (26%)
4 persons	38 (8%)	23 (6%)	151 (17%)	2 (5%)
> 4 persons	8 (2%)	10 (2%)	78 (9%)	2 (5%)
Missing	36	25	90	3
Smoking				
Non-Smoker	169 (38%)	134 (32%)	571 (60%)	19 (46%)
Former smoker	207 (46%)	167 (40%)	260 (27%)	8 (20%)
Smoker	74 (16%)	114 (27%)	128 (13%)	14 (34%)
Missing	35	25	7	0
Alcohol (g/d)	7 (1, 20)	3 (0, 11)	–	–
Missing	63	104	–	–
AUDIT score	–	–	3 (2, 4)	3 (2, 4)
• Missing	–	–	89	7
Physical activity				
Low	42 (10%)	53 (13%)	–	–
Moderate	186 (42%)	167 (41%)	–	–
High	214 (48%)	192 (47%)	–	–
Missing	43	28	–	–
Stress (BiDirect: PSS-14; HaBIDS: PSS-4)	31 (29, 32)	29 (27, 31)	4 (2, 6)	6 (5, 10)
• Missing	1	0	4	0
Intake of Proton pump inhibitors	56 (11%)	101 (22%)	–	–
Missing	5	2	–	–
Chronic lung disease	68 (14%)	99 (22%)	55 (12%)	5 (15%)
Missing	2	1	492	8
Diabetes mellitus	25 (5.0%)	57 (12%)	33 (6%)	4 (11%)
Missing	0	0	444	6
Heart failure	48 (9.5%)	55 (12%)	18 (3%)	1 (3%)
Missing	1	0	442	7
Chronic kidney disease	40 (7.9%)	44 (9.6%)	19 (4%)	0 (0%)
Missing	0	0	500	8
Stroke	11 (2.2%)	21 (4.6%)	11 (2%)	5 (15%)
Missing	0	0	442	7

In BiDirect, all estimates were consistently greater than one (Table 2), albeit some 95% CI included “no association” (i.e. IRR = 1). The p value functions (Online Resource 4) showed that there was the same amount of evidence for “no association” as there was for increased incidence rates (counternull IRR between 1.11 for URTI and 2.18 for fever). In HaBIDS, all estimates except for LRTI and RTI in season 2014/2015 were consistently greater than one (Table 2); the confidence intervals, however, were wide. The p value functions (Online Resource 4) showed that there was the same amount of evidence for “no association” as there was for increased incidence rates of most outcomes (counternull IRR between 1.31 for LRTI and 3.13 for fever) except for RTI in season 2014/2015 (counternull IRR = 0.72) and URTI (counternull IRR = 0.98).

**Table 2:** Adjusted^*^ effect of MDD on infectious diseases in the BiDirect (*n* = 925) and HaBIDS (*n* = 1007) cohort studies

	BiDirect (*n* = 925)	HaBIDS (*n* = 1,007)
Outcome	IRR [95% CI]	IRR [95% CI]
URTI	1.06 [0.93–1.20]	0.99 [0.78–1.26]
LRTI	1.32 [1.00–1.75]	1.15 [0.62–2.14]
Any RTI	1.11 [0.99–1.25]	1.01 [0.79–1.28]
Any RTI in season 2014/2015 (*N* = 751)	–	0.85 [0.59–1.24]
Any RTI in season 2015/2016 (*N* = 689)	–	1.16 [0.85–1.58]
Cystitis	1.12 [0.72–1.76]	1.49 [0.60–3.70]
GI infection	1.68 [1.30–2.16]	1.18 [0.77–1.81]
Fever	1.48 [1.11–1.98]	1.77 [1.10–2.86]
Intake of antibiotics	1.12 [0.89–1.41]	–
Herpes labialis	–	1.35 [0.68–2.65]
Cough lasting more than 4 weeks	–	1.07 [0.56–2.06]

^*^BiDirect: adjusted for age, sex, socioeconomic status (years of education, marital status, income), BMI, household size, smoking, alcohol intake, physical activity, stress, intake of proton pump inhibitors, chronic lung disease, diabetes mellitus, heart failure, chronic kidney disease and stroke

*HaBIDS* adjusted for age, sex, socioeconomic status (education level, marital status, income), household size, smoking, alcohol intake, stress, chronic lung disease, diabetes mellitus, heart failure, chronic kidney disease and stroke

For all outcome variables (except for URTI) we found a dose–response relationship between depressive symptom severity (measured by CES-D) and risk of infection (Table 3), i.e. the IRR increased when depressive symptom severity increased from mild over moderate to severe.

**Table 3:** Adjusted^*^ effect of depressive symptom severity (CES-D score) on infectious diseases in the BiDirect cohort study (*n* = 918)

BiDirect (*n* = 918)		
Outcome	Category (reference: not depressed)	IRR [95% CI]
URTI	Mild	1.00 [0.85–1.18]
	Moderate	1.09 [0.91–1.29]
	Severe	1.04 [0.86–1.26]
LRTI	Mild	0.90 [0.63–1.30]
	Moderate	0.96 [0.65–1.41]
	Severe	1.45 [1.00–2.09]
Any RTI	Mild	0.98 [0.84–1.15]
	Moderate	1.06 [0.90–1.25]
	Severe	1.14 [0.97–1.36]
Cystitis	Mild	1.65 [0.94–2.90]
	Moderate	1.67 [0.90–3.13]
	Severe	2.41 [1.27–4.56]
GI infection	Mild	1.92 [1.40–2.64]
	Moderate	2.09 [1.40–2.94]
	Severe	3.11 [2.18–4.43]
Fever	Mild	1.22 [0.84–1.79]
	Moderate	1.58 [1.06–2.36]
	Severe	1.96 [1.29–2.97]
Intake of antibiotics	Mild	1.24 [0.92–1.67]
	Moderate	1.37 [1.00–1.88]
	Severe	1.88 [1.36–2.60]

^*^adjusted for age, sex, socioeconomic status (years of education, marital status, income), BMI, household size, smoking, alcohol intake, physical activity, stress, intake of proton pump inhibitors, chronic lung disease, diabetes mellitus, heart failure, chronic kidney disease and stroke

Intake of antidepressant medication was consistent with MDD being protective, except for GI infections (Table 4), but 95% CI were widely overlapping.

**Table 4:** Adjusted^*^ effect of antidepressant treatment on infectious diseases in the BiDirect cohort study (*n* = 925)

BiDirect (*n* = 925)		
Outcome	Category (reference: no MDD)	IRR [95% CI]
URTI	MDD without medication	1.15 [0.99–1.34]
	MDD with medication	0.97 [0.83–1.13]
LRTI	MDD without medication	1.44 [1.05–1.98]
	MDD with medication	1.21 [0.87–1.67]
Any RTI	MDD without medication	1.21 [1.06–1.39]
	MDD with medication	1.02 [0.89–1.17]
Cystitis	MDD without medication	1.35 [0.80–2.28]
	MDD with medication	0.89 [0.51–1.53]
GI infections	MDD without medication	1.61 [1.19–2.17]
	MDD with medication	1.75 [1.31–2.36]
Fever	MDD without medication	1.74 [1.25–2.42]
	MDD with medication	1.23 [0.86–1.74]
Intake of antibiotics	MDD without medication	1.23 [0.94–1.61]
	MDD with medication	1.02 [0.78–1.34]

^*^adjusted for age, sex, socioeconomic status (years of education, marital status, income), BMI, household size, smoking, alcohol intake, physical activity, stress, intake of proton pump inhibitors, chronic lung disease, diabetes mellitus, heart failure, chronic kidney disease and stroke

### Quantitative bias analysis

If the specificity of the MDD diagnoses in HaBIDS was 100%, then the corrected estimates with any sensitivity < 100% would be greater than the observed estimates. If both sensitivity and specificity were smaller than 100%, then the corrected estimates would be smaller than the observed estimates.

If all participants with recent CES-D score ≥ 16 points reported too many infections, then the corrected estimates would decrease in magnitude, but would still be consistently above one (Online Resource 5). The same would be true if 50% of all participants with MDD diagnosis reported too many infections (Online Resource 5). Only if 90% of all participants with MDD diagnosis reported too many infections, the corrected estimates of URTI, any RTI, cystitis and intake of antibiotics would change to below one (Online Resource 5).

## Discussion

MDD was associated with an increased incidence of different mild infections and infection-related variables in the BiDirect cohort study as well as in the HaBIDS study. While we found a non-null effect of MDD on the risk of LRTI, this was not true for URTI. Previous studies with similar results [3, 5, 25] had different limitations. While these studies mostly used routine data sources from health insurances companies, health registers or hospitals and therefore focused on severe outcome variables (e.g. mortality, hospitalization) or ICD diagnoses, our study is the first one using self-reported infections as outcomes in a population-based setting. Self-reported acute infections (bronchitis, ear infection, sinus infection, Streptococcal pharyngitis) have so far only been investigated among US college students aged 18–24 years [2]. We were able to show susceptibility to infection in two cohort studies covering a broad age range (15–74 years). This is important because MDD is a chronic disease and changes in the immune system may occur also in advanced age.

We found a monotone dose–response relationship between self-reported infections during the past 12 months and self-reported depressive symptom severity during the past seven days measured by the CES-D. The CES-D covers only the past seven days, but can still be used as a good proxy for MDD because of the chronicity of MDD and the long duration of depressive episodes. The CES-D has a very good validity, discriminating well between individuals with depression and healthy control subjects [13]. Other studies have also investigated the dose–response relationship, but did not use the CES-D score: Adams et al. found a dose–response relationship between feeling exhausted and acute infectious diseases among US college students [2]. Andersson et al. found no linear relationship between the amount of depressive episodes and infection frequency due to the lack of sufficient data of individuals with several depressive episodes and infection diagnosis [3]. Using the CES-D, a more depression-related instrument than measuring exhaustion, we were able to show a monotone relationship between the sum of a broad spectrum of typical depressive symptoms during the past seven days and infection frequency, adding more evidence to answering the question of a possible dose–response relationship between MDD diagnosis and infection.

The analysis of self-reported medication intake during the past four weeks shows an elevated risk of infections in MDD patients both with and without antidepressant medication compared to individuals without MDD; however, infection frequency was reduced in MDD patients with medication compared to MDD patients without medication so that treating MDD with antidepressants seems to reduce the risk of infections. While this effect of antidepressant medication on infectious diseases has not previously been studied, there are several findings of an anti-inflammatory effect of antidepressant treatment on the level of cytokine production [26]. One exception is the frequency of gastrointestinal infections which is higher in MDD patients with antidepressant medication compared to MDD patients without. Since gastrointestinal infections are in general difficult to differentiate from other causes of gastrointestinal symptoms (i.e. diarrhea, vomiting), gastrointestinal side effects of antidepressants might add a source of bias to the self-report of infections.

There are different mechanisms that can explain the effect of depression on infectious diseases. First, a dysregulation of the immune system in individuals with depression has been reported in several studies [27, 28]. A further link between the immune system and depression is reported on the level of the gut microbiome [26]: There are findings of specific bacterial taxa whose changed occurrence is associated with depression as well as with changes of anti-inflammatory signals, e.g. *Coprococcus* bacteria that are active in the dopamine pathway [29]. In addition, other factors as poor self-care in patients with depression or reduced compliance in general medication intake could as well contribute to the observed effects [30].

If our results are replicated in other studies, infection prevention strategies, like vaccination recommendations, might need to be extended to individuals with depression. The German National Public Health Institute (Robert Koch Institute) recommends vaccination against influenza and *Streptococcus pneumoniae* for individuals with various comorbidities, including those with type 2 diabetes [31], who showed in a large matched cohort study an elevated risk of LRTI (1.40 [1.38–1.43]) and pneumonia (1.58 [1.53–1.64]) [32]. These results are on the same level as our findings for LRTI (1.32 [1.00–1.75]), indicating a similar relative risk of LRTI in individuals with depression and diabetes type 2.

### Strengths and limitations

For the first time, this study (i) adjusted for all confounders identified by the systematic approach of a DAG, (ii) describes a monotone dose–response effect and (iii) shows the effect of antidepressant treatment on infection frequency. Although some assumptions encoded in the DAG were not consistent with the data, we decided against changing the DAG post-hoc to avoid “overfitting” the DAG to the specific dataset that the tests were performed on. We present justifications for all arrows in the DAG, and the composition of the MSAS was insensitive to changes in the direction of the few arrows of bidirectional relationships.

The BiDirect cohort study provides data of high quality to examine changes in individuals with MDD. Using the CES-D in addition to the MDD diagnosis from the baseline examination enabled us to show higher risk of infections for depressed individuals with two different definitions of depression. We assume that there is no misclassification of the MDD diagnosis in BiDirect because of the thorough assessment of depressive symptoms in this study. MDD might, however, been misclassified in HaBIDS. Our bias analysis indicates that as long as all non-depressed participants do not self-report any MDD diagnosis (specificity = 100%), misclassification would result in a bias towards null. If misclassification affected both depressed and non-depressed participants, our results would overestimate the true effect. The consistent results in the BiDirect study indicate that misclassification might have been low in HaBIDS.

Using self-reported infections as an outcome variable is both a strength and a limitation of our study. It reduces bias based on differential health seeking behaviour of depressed and non-depressed individuals. Although our results are supported by other studies not using self-reported infections, they might be influenced by information bias. For instance, mood-congruent memory bias describes the fact that individuals with depression recall more negative biographical events than healthy controls [24]. Depression is also associated with memory impairments [33] due to reduced hippocampal volume and other physiological changes [34]. Thus, the number of infections being remembered might be influenced by different effects leading to either higher or lower numbers. Our bias analysis indicates that overreporting of the number of infections by many depressed participants could have resulted in false-positive associations between MDD and some, but not all outcomes; the main findings regarding LRTI, GI infections, and fever cannot be explained by information bias alone.

For all infection variables we used the lower of two numbers in cases where answer possibilities included a range of numbers. That means that our analysis is likely to underestimate the true effect on the infection variables.

While we were able to adjust for most confounders proposed by the DAG in the regression model of BiDirect data, there were several confounders not available in the HaBIDS dataset. Self-selection of the participants, e.g. by loss to follow-up, could have biased our results, but the two studies probably exhibit different mechanisms of selection due to their varying study designs (long study period and in-person examinations in BiDirect versus short study period and online questionnaires in HaBIDS). However, results in both cohorts point into the same direction, underlining the reliability of our findings.

## Conclusions

Depression increases the susceptibility to different mild infectious diseases in two German cohort studies. Further research is needed to replicate this finding and to determine causal immunological pathways in more detail and to differentiate the components of the bidirectional relationship between depression and infection. The inclusion of depression into infection prevention strategies, like vaccination recommendations, should be part of further discussion.

## Supplementary Information

Below is the link to the electronic supplementary material.

Supplementary file1 (PDF 208 KB)

Supplementary file2 (PDF 255 KB)

Supplementary file3 (PDF 337 KB)

Supplementary file4 (PDF 152 KB)

Supplementary file5 (PDF 640 KB)

## Data Availability

The R code is available at zivgitlab.uni-muenster.de/ruebsame/mdd_infection_germany_bidirect_habids. The data used in this analysis are available from the corresponding author upon reasonable request.
