# Supplementary material for: Major depressive disorders increase the susceptibility to self-reported infections in two German cohort studies
Source: Soc Psychiatry Psychiatr Epidemiol. 2022 Jul 5;58(2):277–86. doi: 10.1007/s00127-022-02328-5 (PMC9922209; doi:10.1007/s00127-022-02328-5)
Supplement: Supplementary file 1 — Supplementary file1 (PDF 208 KB) [file 127_2022_2328_MOESM1_ESM.pdf]

Article title
Major depressive disorders increase the susceptibility to self-reported infections in two German cohort studies

Journal name
Social Psychiatry and Psychiatric Epidemiology

Author names and affiliations
Henning Elpers[1], Henning Teismann, PhD[1], Jürgen Wellmann, PhD[1], Klaus Berger, MD[1], André Karch, MD[1], Nicole Rübsamen, PhD[1,*]

[1] Institute of Epidemiology and Social Medicine, University of Münster, Germany.
[*] Corresponding author:
Nicole Rübsamen | Institute of Epidemiology and Social Medicine | University of Münster | Albert-Schweitzer-Campus 1 | 48149 Münster | Germany
clinepi@uni-muenster.de

Online Resource 5: Results of quantitative bias analysis in case of outcome misclassification

	**If all participants with CES-D score ≥ 16 points reported too many infections**	**If 50% of all participants with MDD diagnosis reported too many infections**	**If 90% of all participants with MDD diagnosis reported too many infections**
**Outcome**	**IRR* [95% CI]**	**IRR* [95% CI]**	**IRR* [95% CI]**
URTI	0.96 [0.84–1.10]	0.92 [0.80–1.04]	0.78 [0.68–0.89]
LRTI	1.23 [0.94–1.62]	1.14 [0.87–1.50]	1.07 [0.82–1.41]
Any RTI	1.01 [0.89–1.14]	0.96 [0.85–1.08]	0.82 [0.72–0.93]
Cystitis	1.08 [0.70–1.66]	1.03 [0.66–1.59]	0.86 [0.56–1.33]
GI infection	1.54 [1.20–1.98]	1.52 [1.18–1.96]	1.33 [1.04–1.71]
Fever	1.46 [1.10–1.94]	1.36 [1.02–1.81]	1.22 [0.91–1.61]
Intake of antibiotics	1.03 [0.82–1.29]	0.98 [0.78–1.22]	0.87 [0.69–1.09]

*adjusted for age, sex, socioeconomic status (years of education, marital status, income), BMI, household size, smoking, alcohol intake, physical activity, stress, intake of proton pump inhibitors, chronic lung disease, diabetes mellitus, heart failure, chronic kidney disease and stroke.
