# Supplementary material for: Major depressive disorders increase the susceptibility to self-reported infections in two German cohort studies
Source: Soc Psychiatry Psychiatr Epidemiol. 2022 Jul 5;58(2):277–86. doi: 10.1007/s00127-022-02328-5 (PMC9922209; doi:10.1007/s00127-022-02328-5)
Supplement: Supplementary file 2 — Supplementary file2 (PDF 255 KB) [file 127_2022_2328_MOESM2_ESM.pdf]

Article title
Major depressive disorders increase the susceptibility to self-reported infections in two German cohort studies

Journal name
Social Psychiatry and Psychiatric Epidemiology

Author names and affiliations
Henning Elpers[1], Henning Teismann, PhD[1], Jürgen Wellmann, PhD[1], Klaus Berger, MD[1], André Karch, MD[1], Nicole Rübsamen, PhD[1,*]

1 Institute of Epidemiology and Social Medicine, University of Münster, Germany.
* Corresponding author:
Nicole Rübsamen | Institute of Epidemiology and Social Medicine | University of Münster | Albert-Schweitzer-Campus 1 | 48149 Münster | Germany
clinepi@uni-muenster.de

Online Resource 1: Directed acyclic graph (DAG) representing assumed causal relationsships between MDD diagnosis (exposure), self-reported infections (outcome), and other variables (possible confounders)

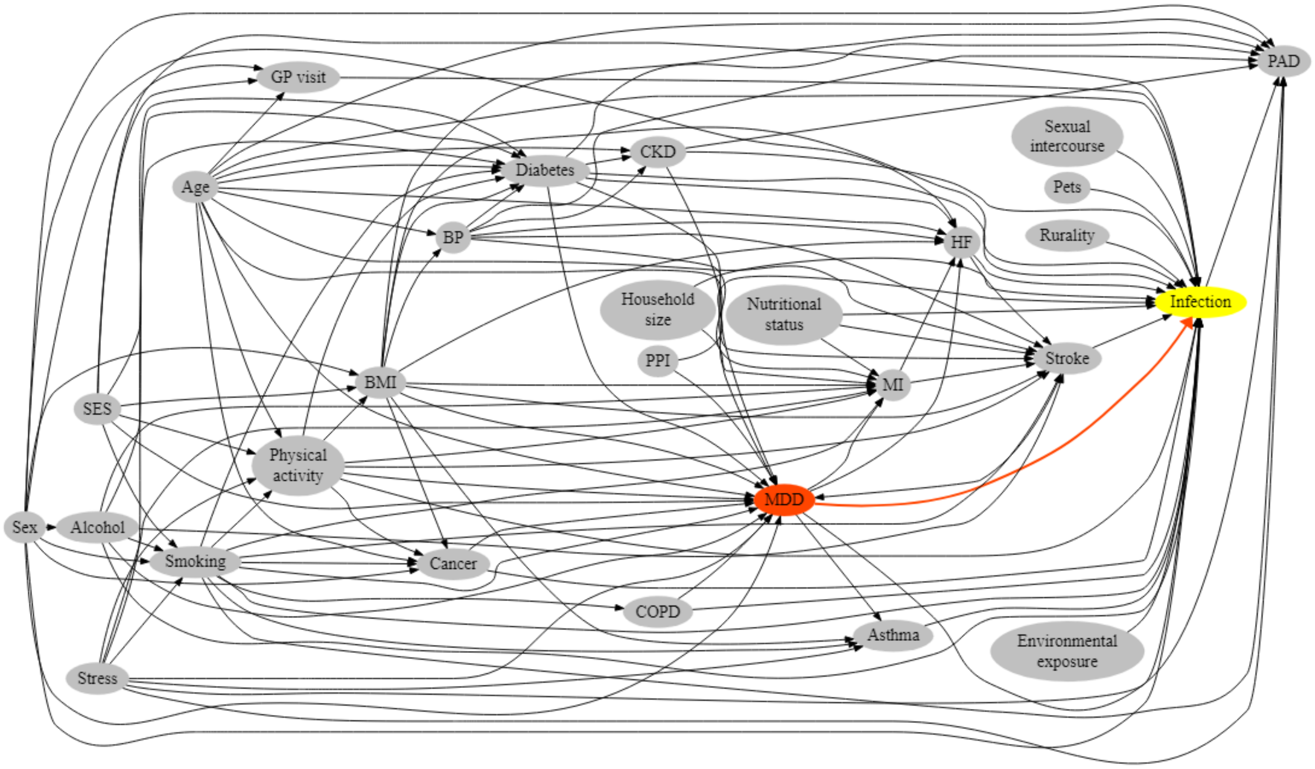

We aim to estimate the direct effect (red arrow) of MDD (exposure, red) on infections (outcome, yellow). Justifications for the arrows defined the DAG are given in Online Resource 2.
