# Supplementary material for: Major depressive disorders increase the susceptibility to self-reported infections in two German cohort studies
Source: Soc Psychiatry Psychiatr Epidemiol. 2022 Jul 5;58(2):277–86. doi: 10.1007/s00127-022-02328-5 (PMC9922209; doi:10.1007/s00127-022-02328-5)
Supplement: Supplementary file 3 — Supplementary file3 (PDF 337 KB) [file 127_2022_2328_MOESM3_ESM.pdf]

Article title
Major depressive disorders increase the susceptibility to self-reported infections in two German cohort studies

Journal name
Social Psychiatry and Psychiatric Epidemiology

Author names and affiliations
Henning Elpers[1], Henning Teismann, PhD[1], Jürgen Wellmann, PhD[1], Klaus Berger, MD[1], André Karch, MD[1], Nicole Rübsamen, PhD[1,*]

[1] Institute of Epidemiology and Social Medicine, University of Münster, Germany.
[*] Corresponding author:
Nicole Rübsamen | Institute of Epidemiology and Social Medicine | University of Münster | Albert-Schweitzer-Campus 1 | 48149 Münster | Germany
clinepi@uni-muenster.de

Online Resource 2: Justifications for the arrows defined the directed acyclic graph

X	causes	Y	Type	References	Quote	Comment
Age	→	MDD		Hasin et al. 2005; Fiske et al. 2009	Middle age as risk factor for depression	
Age	→	Infection	CAP	Almirall et al. 2017	aOR 1.07 (1.01–1.19) per year of increase	
Age	→	Infection	CAP	Torres et al. 2013	The incidence of CAP increased with age […].	
Age	→	Infection	ARI	Chen et al. 2014	Age was an independent risk factor for ARI, with the risk of acquiring ARI decreasing as age increased.	
Age	→	Infection	AGE	Ecollan et al. 2020	[…] compared to individuals between 15 and 60 yo, elderly (≥ 60 yo) tend to have fewer AGE episodes (aOR = 0.76, 95% CI [0.64; 0.89]), as children between 10 and 15 y (aOR = 0.60 [0.37; 0.98]).	
Sex	→	MDD		Radloff 1975	In the total sample, women are more depressed than men.	
Sex	→	Infection	RTI	Falagas et al. 2007	Overall, it seems that males develop RTIs more frequently than females, except for sinusitis, otitis externa, and probably tonsillitis.	

X	causes	Y	Type	References	Quote	Comment
Sex	→	Infection	ARI	Chen et al. 2014	Gender was found to be associated with ARI in the separate logistic model, with males having a lower risk of experiencing respiratory infections compared to females (P = 0.023).	
Sex	→	Infection	CAP	Torres et al. 2013	Overall CAP incidence and hospitalisation for CAP were higher in men than in women.	
Sex	not	Infection	CAP	Almirall et al. 2017	No definite conclusion	
BMI	→	MDD		Roberts et al. 2003	Obesity at baseline was associated with increased risk of depression 5 y later […]	
BMI	→	Infection	CAP	Falagas & Kompoti 2006	[…] the BMI was shown to be directly associated with an increased risk of community-acquired pneumonia among women.	
BMI	→	Infection	CAP	Torres et al. 2013	Being underweight was generally associated with an increased risk of CAP (crude ORs: 1.04 (0.57 to 1.89) to 2.20 (1.57 to 3.09)) compared with normal bodyweight (OR 1.00). A reduced risk was seen in individuals classified as overweight (crude ORs: 0.6 (0.5 to 0.7) to 0.89 (0.72 to 1.09) […].	
BMI	not	Infection	CAP	Almirall et al. 2017	No effect	
BMI	→	Infection	URTI/LRTI	Maccioni et al. 2018	Obesity was associated both with lower RTIs (adjusted OR = 2.02, 95%CI = 1.36–3.00) and upper RTIs (adjusted OR = 1.55, 95%CI = 1.22–1.96).	
BMI	→	Infection	AGE	Ecollan et al. 2020	We also highlight three health characteristics associated with AGE: overweight (aOR = 1.25 [1.07;1.45]) and obesity (aOR = 1.47 [1.19;1.81]) […].	
SES	→	MDD		Everson et al. 2002	Several studies have found that major depressive disorder and greater depressive symptomatology are more prevalent at lower levels of SES.	
SES	→	BMI		Everson et al. 2002	[…] several sources indicate that rates of obesity are higher among lower SES individuals.	

X	causes	Y	Type	References	Quote	Comment
SES	→	Diabetes		Everson et al. 2002	Numerous studies have found an inverse relationship between Type II diabetes and education, occupation, and income that is consistent across all adult age groups.	
SES	→	Infection	CAP	Torres et al. 2013	Higher levels of education were associated with a lower risk of CAP.	
Household size	→	MDD		Stahl et al. 2017	Living alone (compared to living with a family member) was associated with elevated levels of depressive symptomatology.	
Household size	→	Infection	ARI	Chen et al. 2014	We observed dose response of increasing odds for increasing household size.	
Household size	→	Infection	CAP	Torres et al. 2013	Living in a household of over 10 people was associated with a crude OR of 2.20 (1.21 to 4.00).	
Household size	→	Infection	AGE	Ecollan et al. 2020	Living alone is also associated with having AGE episode (aOR = 1.31 [1.09; 1.59]) compared to people living with adults.	
Rurality	→	Infection	ARI	Chen et al. 2014	People from rural communities were less likely to report ARI compared with people living in major cities (P=0.001).	
Smoking	→	MDD		Flensborg-Madsen et al. 2011	The study suggests that smoking is associated with increased risk of developing depression.	
Smoking	→	Infection	CAP	Torres et al. 2013	There was consistent evidence that smoking was associated with an increased risk of CAP.	
Smoking	→	Infection	CAP	Almirall et al. 2017	Adjusted odds ratio: 1.57 (1.12 – 2.77) vs. never smokers	
Alcohol	→	MDD		Boden & Fergusson 2011	Further evidence suggests that the most plausible causal association between alcohol use disorders (AUD) and major depression (MD) is one in which AUD increases the risk of MD, rather than vice versa.	

X	causes	Y	Type	References	Quote	Comment
Alcohol	→	Infection	CAP	Torres et al. 2013	Compared with individuals who consumed no alcohol (OR 1.00), consumption of ≤40 g alcohol daily appeared to protect against CAP (21–40 g/day, crude ORs: 0.53 (0.22 to 1.25) and 0.88 (0.63 to 1.22)). However, the risk increased in individuals with higher consumption (>41 g/day, crude OR: 1.59 (0.59 to 4.25)23; >80 g/day, crude OR: 2.34 (1.13 to 4.85)) or with a history of alcohol abuse/alcoholism (crude ORs: 1.85 (1.19 to 2.88)21 and 1.62 (0.91 to 2.91)).	
Alcohol	not	Infection	CAP	Almirall et al. 2017	No definite conclusion	
GP visit	→	Infection	CAP	Torres et al. 2013	[…] one study found that frequent visits to the general practitioner in the previous year were associated with a substantial increase in the risk of CAP (1–4 visits, OR 1.00; ≥30 visits, crude OR 3.73 (3.14 to 4.42)).	also check "Infection → GP visit"
Environmental exposure	→	Infection	CAP	Almirall et al. 2017	Clear risk factor	
Poor nutritional status	→	Infection	CAP	Almirall et al. 2017	aOR 6.14 (0.65-11.58)	
Stress	→	MDD		Plieger et al. 2015	A significantly closer relation between depression and life stress than between burnout and life stress was found in the healthy (z = 3.01, p = .003) as well as in the depressed sample (z = 3.41, p = .001).	
Stress	→	Infection	ARI	Falagas et al. 2010	Individuals with higher levels of perceived stress or daily stress […] are more susceptible to acute infections of the respiratory tract than individuals without these traits.	
Physical activity	→	MDD		Teychenne et al. 2008	Although the dose and domain of physical activity (PA) varied across studies reviewed, evidence suggests that even low doses of PA may be protective against depression.	
Physical activity	not	Infection	URTI	Ghilotti et al. 2018	We did not find any association between physical activity, sleep duration or sleep quality and the occurrence of upper respiratory tract infections in adult Swedish population.	

X	causes	Y	Type	References	Quote	Comment
Physical activity	→	Infection	URTI	Fondell et al. 2011	We found that high levels of physical activity (≥55 MET×h×d-1) were associated with an 18% reduced risk (incidence rate ratio (IRR) = 0.82, 95% confidence interval (CI) = 0.69–0.98) of self-reporting URTI compared with low levels of physical activity (<45 MET×h×d-1).	
SES	→	Smoking		Hiscock et al. 2012	Smoking rates are higher among those with lower SES in the majority of developed countries […]	
HF	→	Infection	CAP	Torres et al. 2013	Chronic cardiovascular disease increased the risk of CAP up to threefold (crude ORs from 1.4 (1.2 to 1.5) to 3.2 (2.6 to 4.1)). Additional studies supported an association between chronic heart disease (adjusted ORs: 1.63 (1.54 to 1.72) and 1.66 (1.59 to 1.73)) or heart failure (adjusted ORs: 2.19 (0.69 to 6.95) and 1.37 (1.20 to 1.57); adjusted RR: 2.63 (2.21 to 3.14)) and the risk of CAP.	
MDD	→	MI		Van der Kooy et al. 2007	Depressed mood moderately increased the risk for MI, CHD, cerebrovascular diseases and other CVDs to the same level (1.43–1.63).	
MDD	→	HF		Celano et al. 2018	Both depression and anxiety disorders are associated with the development and progression of HF […].	
Chronic diseases	not	Infection	ARI	Chen et al. 2014	Respondents with heart disease, arthritis, hypertension and cancer were found less likely to report ARI in the separate logistic model. However, this does not mean that these chronic conditions are protective factors for ARI; as age was strongly associated with chronic diseases, for example heart disease (data not shown), and age was also found to be a significant predictor for ARI, and therefore the detected associations between those diseases and ARI were confounded by age.	
MDD	→	PAD		Ramirez et al. 2018	Current evidence strongly suggests a relationship between depression and PAD, and depression has been linked to poor outcomes in patients with PAD.	
Stroke	→	Infection	CAP	Torres et al. 2013	Cerebrovascular disease/stroke and dementia approximately doubled the risk of CAP […]	
Stroke	→	MDD		Chemerinski & Robinson 2000	Depression is among the most common neuropsychiatric disorders occurring after stroke.	also check

X	causes	Y	Type	References	Quote	Comment
						"MDD → Stroke" (Jonas & Mussolino 2000)
Asthma	→	Infection	ARI	Chen et al. 2014	A marked association was identified between asthma and ARI (P=0.001) after controlling for potential confounding in the final logistic model, and respondents with asthma were more likely to experience ARI compared with respondents without asthma (OR= 1.41).	
Asthma	→	Infection	CAP	Torres et al. 2013	Patients with chronic respiratory diseases, including COPD, bronchitis or asthma, had a twofold to fourfold increase in the risk of CAP […].	
MDD	→	Asthma		Gao et al. 2015	Depression was associated with a 43% increased risk of developing adult-onset asthma.	
Asthma	not	MDD		Gao et al. 2015	However, asthma did not increase the risk of depression based on limited studies.	
COPD	→	Infection	LRTI	Sethi 2010	The lung in COPD becomes susceptible to repeated acute airway mucosal infections with viruses and bacterial pathogens, leading to episodes of increased inflammation and worsened symptoms, which are clinically diagnosed as exacerbations of COPD.	
COPD	→	MDD		Schneider et al. 2010	This large observational study provides further evidence that patients with COPD are at an increased risk of developing depression.	
Diabetes	→	Infection		Peleg et al. 2007	The weight of evidence suggests that diabetes is a risk factor for serious respiratory infections […]	
Diabetes	→	MDD		Renn et al. 2011	The biochemical and physiological changes associated with diabetes, as well as the psychosocial burden of a chronic disease, lend modest evidence to the argument that depression is a consequence of diabetes.	
CKD	→	Infection		McDonald et al. 2014	Predialysis kidney disease appears to be associated with increased risk of severe infection.	
CKD	→	MDD		Palmer et al. 2013	Approximately 20%-30% of people with severe CKD may fulfill the criteria for a depression diagnosis, although identifying depression may be more difficult in patients with CKD due to the coexistence of somatic symptoms.	

X	causes	Y	Type	References	Quote	Comment
Cancer	→	Infection	LRTI	Hijano et al. 2018	In immunocompromised adults with cancer, progression to LRTI ranges from 30 to 50%, and for those who develop pneumonia, mortality can be as high as 75%.	
Cancer	→	Infection	CAP	Torres et al. 2013	Cancer was also associated with a moderate increase in the risk of CAP […]	
Cancer	→	MDD		Mitchell et al. 2011	Our results suggest that in the first 5 years after diagnosis about a sixth of people with cancer have syndromal depression, rising to about a third with either depression or adjustment disorder.	also check "MDD → Cancer" (Jia et al. 2017)
Stress	→	Physical activity		Stults-Kolehmainen & Sinha 2014	Overall, the majority of the literature finds that the experience of stress impairs efforts to be physically active.	also check "Physical activity → Stress" (Vankim & Nelson 2013)
Stress	→	Physical activity		Ng & Jeffery 2003	High stress for both men and women was associated with a higher fat diet, less frequent exercise […]	
SES	→	Physical activity		Lindström et al. 2001	Lower socioeconomic groups had a higher risk of being in the lower quartile of leisure-time physical activity.	
Smoking	→	Physical activity		Kaczynski et al. 2008	Overall, smoking and PA appear to be negatively associated, but such generalizations must be made with caution for several reasons.	
Smoking	→	Physical activity		Paavola et al. 2004	Smoking correlated negatively with leisure-time physical activity.	
Alcohol	→	Smoking		Paavola et al. 2004	Smoking and alcohol use correlated positively for each survey	also check "Smoking → Alcohol"
Stress	→	Smoking		Ng & Jeffery 2003	High stress for both men and women was associated with a higher fat diet, less frequent exercise, cigarette smoking […]	
Sex	→	Smoking		Pampel 2006	Western Europe: % female smokers = 23.4; % male smokers = 30.1	
Sex	→	Alcohol		Erol & Karpyak 2015	[…] non-alcoholic women consume less alcohol compared to non-alcoholic men and are less prone to behavioral problems associated with acute alcohol consumption.	
Sex	→	BMI		Lovejoy & Sainsbury 2009	The prevalence of obesity is higher in women than in men in most countries around the world	

X	causes	Y	Type	References	Quote	Comment
Age	→	Diabetes		Pan et al. 1997	On average, subjects with diabetes are older, have higher personal annual incomes, and more often have a family history of diabetes. They also have higher mean BMI, waist-to-hip ratio (WHR), systolic blood pressure, diastolic blood pressure, and a greater prevalence of hypertension. They perform less physical activity and have less education than people with normal oral glucose tolerance test results.	
SES	→	Diabetes		Pan et al. 1997	On average, subjects with diabetes are older, have higher personal annual incomes, and more often have a family history of diabetes. They also have higher mean BMI, waist-to-hip ratio (WHR), systolic blood pressure, diastolic blood pressure, and a greater prevalence of hypertension. They perform less physical activity and have less education than people with normal oral glucose tolerance test results.	
BMI	→	Diabetes		Pan et al. 1997	On average, subjects with diabetes are older, have higher personal annual incomes, and more often have a family history of diabetes. They also have higher mean BMI, waist-to-hip ratio (WHR), systolic blood pressure, diastolic blood pressure, and a greater prevalence of hypertension. They perform less physical activity and have less education than people with normal oral glucose tolerance test results.	
BP	→	Diabetes		Pan et al. 1997	On average, subjects with diabetes are older, have higher personal annual incomes, and more often have a family history of diabetes. They also have higher mean BMI, waist-to-hip ratio (WHR), systolic blood pressure, diastolic blood pressure, and a greater prevalence of hypertension. They perform less physical activity and have less education than people with normal oral glucose tolerance test results.	
Physical activity	→	Diabetes		Pan et al. 1997	On average, subjects with diabetes are older, have higher personal annual incomes, and more often have a family history of diabetes. They also have higher mean BMI, waist-to-hip ratio (WHR), systolic blood pressure, diastolic blood pressure, and a greater prevalence of hypertension. They perform less physical activity and have less education than people with normal oral glucose tolerance test results.	

X	causes	Y	Type	References	Quote	Comment
BP	→	Stroke		Barrett-Connor & Khaw 1988	Raised blood pressure is the strongest single risk factor for stroke in the general population.	
Diabetes	→	Stroke		Barrett-Connor & Khaw 1988	These findings support the hypothesis that diabetes may confer excess risk of stroke independent of blood pressure.	
Stress	→	Diabetes		Hackett & Steptoe 2017	Accumulating evidence, for the most part, has linked various psychological stress factors with new onset T2DM.	
Alcohol	→	Diabetes		Baliunas et al. 2009	Our meta-analysis confirms the U-shaped relationships between average amount of alcohol consumed per day and risk of incident type 2 diabetes among men and women, although a more protective effect of moderate consumption was found for women.	
Smoking	→	Diabetes		Eliasson 2003	Prospectively, the increased risk for diabetes in smoking men and women is around 50%.	
Sex	→	PAD		Criqui & Aboyans 2015	Prevalence seems to be higher among men than women for more severe or symptomatic disease.	
Smoking	→	PAD		Criqui & Aboyans 2015	Smoking is one of the strongest risk factors for PAD in virtually all studies.	
Diabetes	→	PAD		Criqui & Aboyans 2015	Diabetes mellitus is strongly associated with an elevated risk of PAD.	
BP	→	PAD		Criqui & Aboyans 2015	The association of hypertension with PAD has been demonstrated in most studies in which blood pressure was studied.	
BMI	→	PAD		Criqui & Aboyans 2015	To date, the preponderance of evidence fails to support a consistent, independent positive association between obesity and PAD.	
Alcohol	→	PAD		Criqui & Aboyans 2015	Evidence for a protective effect of light-to-moderate alcohol consumption, as seen in coronary heart disease (CHD), is less consistent for PAD.	
CKD	→	PAD		Criqui & Aboyans 2015	Several studies have shown an association between chronic kidney disease defined according to creatinine levels and PAD […]	

X	causes	Y	Type	References	Quote	Comment
Infection	→	PAD		Criqui & Aboyans 2015	A study in young women found that self-reported history of various types of infectious diseases, such as chicken pox, shingles, mumps, pneumonia, chronic bronchitis, or peptic ulcer, was independently and significantly related to PAD.	
Stress	→	PAD		Criqui & Aboyans 2015	Psychosocial factors were found to be associated with PAD […]	
BP	→	Stroke		Rundek & Sacco 2008	Hypertension is one of the most important modifiable risk factors for prevention of a first stroke.	
Diabetes	→	Stroke		Rundek & Sacco 2008	Individuals with type 2 diabetes are considered at high risk for vascular events […]	
MI	→	Stroke		Rundek & Sacco 2008	Other types of cardiac disease that can contribute to the risk of thromboembolic stroke include myocardial infarction […]	
HF	→	Stroke		Rundek & Sacco 2008	Independent stroke predictors included in the Framingham Stroke Profile are age, systolic blood pressure, hypertension, diabetes mellitus, current smoking, established cardiovascular disease (any one of myocardial infarction, angina or coronary insufficiency, congestive heart failure, or intermittent claudication), atrial fibrillation, and left ventricular hypertrophy on EKG.	
Smoking	→	Stroke		Rundek & Sacco 2008	Cigarette smoking is a well-recognized and modifiable risk factor for ischemic and hemorrhagic stroke.	
Poor nutritional status	→	Stroke		Rundek & Sacco 2008	Diet is associated with the risk of stroke. Increased fruit and vegetable consumption is associated with a reduced risk of stroke in a dose-response manner.	
Physical activity	→	Stroke		Rundek & Sacco 2008	Physical inactivity is a well-established and modifiable risk factor for stroke.	
BMI	→	Stroke		Rundek & Sacco 2008	Obesity is a risk factor for stroke (relative risks, 1.5−2.0) and is associated with increased risk of hypertension, dyslipidemia, hyperinsulinemia, and glucose intolerance.	

X	causes	Y	Type	References	Quote	Comment
BMI	→	BP		Rundek & Sacco 2008	Obesity is a risk factor for stroke (relative risks, 1.5−2.0) and is associated with increased risk of hypertension, dyslipidemia, hyperinsulinemia, and glucose intolerance.	
Alcohol	→	Stroke		Rundek & Sacco 2008	The J-shaped relation of alcohol consumption to the risk of stroke has been reported.	
Age	→	HF		Meijers & de Boer 2019	Although differences between these models exist, most risk models have reported a quite consistent list of risk factors including age, sex, coronary artery disease (CAD), myocardial infarction (MI), hypertension, diabetes mellitus, and obesity as the most important risk factors.	
Sex	→	HF		Meijers & de Boer 2019	Not so long ago, a typical HF patient was male and had endured a MI. However, the last years, women are catching up: they tend to develop HF at a more advanced age, present more often with HF with preserved ejection fraction, are more symptomatic, and tend to have more comorbidities than men.	
MI	→	HF		Meijers & de Boer 2019	Even though identification and treatment of patients with MI has improved significantly the past decade, it still remains the most important risk factor for incident HF.	
BP	→	HF		Meijers & de Boer 2019	Hypertension is a prevalent and a well-established risk factor for HF.	
Diabetes	→	HF		Meijers & de Boer 2019	Patients with diabetes mellitus have an increased risk of developing HF […]	
BMI	→	HF		Meijers & de Boer 2019	While the risk of developing HF has been shown to be higher in patients who are obese, a survival advantage exists for overweight/obese patients in comparison to average weight patients.	
Age	→	Cancer		Meijers & de Boer 2019	Established risk factors are race, age, sex, genetics, body mass index, family history of cancer, history of tobacco use, lack of physical activity […]	
Sex	→	Cancer		Meijers & de Boer 2019	Men are more likely to be diagnosed with HF at an earlier age, as discussed above, and they are also more frequently diagnosed with cancer.	

X	causes	Y	Type	References	Quote	Comment
BMI	→	Cancer		Meijers & de Boer 2019	It is speculated that 20% of the incidence of cancer is related to obesity.	
Smoking	→	Cancer		Meijers & de Boer 2019	There is massive evidence on the unequivocal relation between smoking and cancer.	
Physical activity	→	Cancer		Meijers & de Boer 2019	Established risk factors are race, age, sex, genetics, body mass index, family history of cancer, history of tobacco use, lack of physical activity […]	
Smoking	→	MI		Yusuf et al. 2004	Smoking (odds ratio 2.87 for current vs never, population attributable risks (PAR) 35.7% for current and former vs never) […]	
Blood pressure	→	MI		Yusuf et al. 2004	[…] history of hypertension (1.91, PAR 17.9%) […]	
BMI	→	MI		Yusuf et al. 2004	[…] abdominal obesity (1.12 for top vs lowest tertile and 1.62 for middle vs lowest tertile, PAR 20.1% for top two tertiles vs lowest tertile) […]	
Stress	→	MI		Yusuf et al. 2004	[…] psychosocial factors (2.67, PAR 32.5%) […]	
Poor nutritional status	→	MI		Yusuf et al. 2004	[…] daily consumption of fruits and vegetables (0.70, PAR 13.7% for lack of daily consumption) […]	
Alcohol	→	MI		Yusuf et al. 2004	[…] regular alcohol consumption (0.91, PAR 6.7%) […]	
Physical activity	→	MI		Yusuf et al. 2004	[…] regular physical activity (0.86, PAR 12.2%) […]	
Smoking	→	Asthma		Toskala & Kennedy 2015	Active smoking has been shown to be risk factor for developing asthma […]	
BMI	→	Asthma		Toskala & Kennedy 2015	In addition to increasing the risks of the respiratory effects of air pollution, obesity alone has a significant impact on asthma risk, phenotype, and prognosis.	
Stress	→	Asthma		Toskala & Kennedy 2015	Current evidence suggests a causal association between chronic psychosocial stress and asthma or asthma morbidity.	
Smoking	→	COPD		Vijayan 2013	Tobacco smoking is the main cause of obstructive pulmonary disease.	
Diabetes	→	CKD		Ammirati 2020	The main causes of CKD include diabetes, hypertension […]	

X	causes	Y	Type	References	Quote	Comment
BP	→	CKD		Ammirati 2020	The main causes of CKD include diabetes, hypertension […]	
SES	→	GP visit		Hoebel et al. 2016	[…] outpatients with low SES had more contacts with general practitioners than outpatients with high SES (men: incidence rate ratio [IRR] = 1.25; 95% confidence interval [CI] = 1.08–1.46; women: IRR = 1.20; 95% CI = 1.07–1.34).	
Sex	→	GP visit		Corney 1990	Women's rates of utilization of almost all health care services are higher than men's. General practice surveys endorse these findings with women attending more frequently […]	
PPI	→	MDD		Novotny et al. 2018	The findings of most research studies described in this review indicate that there is a direct association between the onset of dementia and depression on one side and the long-term use of PPIs on the other.	
PPI	→	Infection	AGE	Chen et al. 2016	PPI use is associated with an increased risk of infectious gastroenteritis hospitalization.	
Pets	→	Infection	AGE	Ecollan et al. 2020	Having pets at home is associated with having AGE episode (aOR = 1.23 [1.08; 1.41]).	
Sexual intercourse	→	Infection	urinary tract infection	Hooton et al. 1996	Among sexually active young women the incidence of symptomatic urinary tract infection is high, and the risk is strongly and independently associated with recent sexual intercourse, recent use of a diaphragm with spermicide, and a history of recurrent urinary tract infections.	

Abbreviations

AGE	Acute gastroenteritis
aOR	Adjusted odds ratio
ARI	Acute respiratory infection
BMI	Body mass index
BP	Blood pressure
CAP	Community-acquired pneumonia
CKD	Chronic kidney disease
COPD	Chronic obstructive pulmonary disease
GP	General practitioner
HF	Heart failure
LRTI	Lower respiratory tract infection

MDD Major depressive disorders
MI Myocardial infarction
PAD Peripheral artery disease
PPI Proton-pump inhibitors
RTI Respiratory tract infection
SES Socioeconomic status
URTI Upper respiratory tract infection

References

- Almirall J, Serra-Prat M, Bolíbar I, Balasso V. 2017. Risk Factors for Community-Acquired Pneumonia in Adults: A Systematic Review of Observational Studies. Respiration; international review of thoracic diseases 94: 299–311. doi: 10.1159/000479089
- Ammirati AL. 2020. Chronic Kidney Disease. Revista da Associacao Medica Brasileira (1992) 66Suppl 1: s03–s09. doi: 10.1590/1806-9282.66.S1.3
- Baliunas DO, Taylor BJ, Irving H, Roerecke M, Patra J, Mohapatra S, Rehm J. 2009. Alcohol as a risk factor for type 2 diabetes: A systematic review and meta-analysis. Diabetes Care 32: 2123–2132. doi: 10.2337/dc09-0227
- Barrett-Connor E, Khaw K-T. 1988. DIABETES MELLITUS: AN INDEPENDENT RISK FACTOR FOR STROKE? American Journal of Epidemiology 128: 116–123. doi: 10.1093/oxfordjournals.aje.a114934
- Boden JM, Fergusson DM. 2011. Alcohol and depression. Addiction 106: 906–914. doi: 10.1111/j.1360-0443.2010.03351.x
- Celano CM, Villegas AC, Albanese AM, Gaggin HK, Huffman JC. 2018. Depression and Anxiety in Heart Failure: A Review. Harvard review of psychiatry 26: 175–184. doi: 10.1097/HRP.0000000000000162
- Chemerinski E, Robinson RG. 2000. The neuropsychiatry of stroke. Psychosomatics 41: 5–14. doi: 10.1016/S0033-3182(00)71168-6
- Chen Y, Liu B, Glass K, Du W, Banks E, Kirk M. 2016. Use of Proton Pump Inhibitors and the Risk of Hospitalization for Infectious Gastroenteritis. PloS one 11: e0168618. doi: 10.1371/journal.pone.0168618
- Chen Y, Williams E, Kirk M. 2014. Risk factors for acute respiratory infection in the Australian community. PLoS ONE 9: 1–7. doi: 10.1371/journal.pone.0101440
- Corney RH. 1990. Sex differences in general practice attendance and help seeking for minor illness. Journal of psychosomatic research 34: 525–534. doi: 10.1016/0022-3999(90)90027-2
- Criqui MH, Aboyans V. 2015. Epidemiology of peripheral artery disease. Circulation research 116: 1509–1526. doi: 10.1161/CIRCRESAHA.116.303849
- Ecollan M, Guerrisi C, Souty C, Rossignol L, Turbelin C, Hanslik T, Colizza V, Blanchon T. 2020. Determinants and risk factors of gastroenteritis in the general population, a web-based cohort between 2014 and 2017 in France. BMC Public Health 20: 1146. doi: 10.1186/s12889-020-09212-4
- Eliasson B. 2003. Cigarette smoking and diabetes. Progress in Cardiovascular Diseases 45: 405–413. doi: https://doi.org/10.1053/pcad.2003.00103
- Erol A, Karpyak VM. 2015. Sex and gender-related differences in alcohol use and its consequences: Contemporary knowledge and future research considerations. Drug and Alcohol Dependence 156: 1–13. doi: https://doi.org/10.1016/j.drugalcdep.2015.08.023
- Everson SA, Maty SC, Lynch JW, Kaplan GA. 2002. Epidemiologic evidence for the relation between socioeconomic status and depression, obesity, and diabetes. Journal of Psychosomatic Research 53: 891–895. doi: https://doi.org/10.1016/S0022-3999(02)00303-3
- Falagas ME, Karamanidou C, Kastoris AC, Karlis G, Rafailidis PI. 2010. Psychosocial factors and susceptibility to or outcome of acute respiratory tract infections. International Journal of Tuberculosis and Lung Disease 14: 141–148.
- Falagas ME, Kompoti M. 2006. Obesity and infection. The Lancet Infectious Diseases 6: 438–446. doi: https://doi.org/10.1016/S1473-3099(06)70523-0
- Falagas ME, Mourtzoukou EG, Vardakas KZ. 2007. Sex differences in the incidence and severity of respiratory tract infections. Respiratory Medicine 101: 1845–1863. doi: https://doi.org/10.1016/j.rmed.2007.04.011

- Fiske A, Wetherell JL, Gatz M. 2009. Depression in Older Adults. Annual Review of Clinical Psychology 5: 363–389. doi: 10.1146/annurev.clinpsy.032408.153621
- Flensborg-Madsen T, Bay von Scholten M, Flachs EM, Mortensen EL, Prescott E, Tolstrup JS. 2011. Tobacco smoking as a risk factor for depression. A 26-year population-based follow-up study. Journal of Psychiatric Research 45: 143–149. doi: https://doi.org/10.1016/j.jpsychires.2010.06.006
- Fondell E, Lagerros YT, Sundberg CJ, Lekander M, Bälter O, Rothman KJ, Rothman KJ, Bälter K. 2011. Physical activity, stress, and self-reported upper respiratory tract infection. Medicine and science in sports and exercise 43: 272–279. doi: 10.1249/mss.0b013e3181edf108
- Gao YH, Zhao HS, Zhang FR, Gao Y, Shen P, Chen R, Zhang GJ. 2015. The relationship between depression and asthma: A meta-analysis of prospective studies. PLoS ONE 10: 1–12. doi: 10.1371/journal.pone.0132424
- Ghilotti F, Pesonen AS, Raposo SE, Winell H, Nyrén O, Lagerros YT, Plymoth A. 2018. Physical activity, sleep and risk of respiratory infections: A Swedish cohort study. PLoS ONE 13: 1–12. doi: 10.1371/journal.pone.0190270
- Hackett RA, Steptoe A. 2017. Type 2 diabetes mellitus and psychological stress-a modifiable risk factor. Nature Reviews Endocrinology 13: 547–560. doi: 10.1038/nrendo.2017.64
- Hasin DS, Goodwin RD, Stinson FS, Grant BF. 2005. Epidemiology of major depressive disorder: results from the National Epidemiologic Survey on Alcoholism and Related Conditions. Archives of general psychiatry 62: 1097–1106. doi: 10.1001/archpsyc.62.10.1097
- Hijano DR, Maron G, Hayden RT. 2018. Respiratory Viral Infections in Patients With Cancer or Undergoing Hematopoietic Cell Transplant. Frontiers in Microbiology 9: 3097.
- Hiscock R, Bauld L, Amos A, Fidler JA, Munafò M. 2012. Socioeconomic status and smoking: A review. Annals of the New York Academy of Sciences 1248: 107–123. doi: 10.1111/j.1749-6632.2011.06202.x
- Hoebel J, Rattay P, Prütz F, Rommel A, Lampert T. 2016. Socioeconomic Status and Use of Outpatient Medical Care: The Case of Germany. PloS one 11: e0155982. doi: 10.1371/journal.pone.0155982
- Hooton TM, Scholes D, Hughes JP, Winter C, Roberts PL, Stapleton AE, Stergachis A, Stamm WE. 1996. A prospective study of risk factors for symptomatic urinary tract infection in young women. The New England journal of medicine 335: 468–474. doi: 10.1056/NEJM199608153350703
- Jia Y, Li F, Liu YF, Zhao JP, Leng MM, Chen L. 2017. Depression and cancer risk: a systematic review and meta-analysis. Public Health 149: 138–148. doi: https://doi.org/10.1016/j.puhe.2017.04.026
- Jonas BS, Mussolino ME. 2000. Symptoms of Depression as a Prospective Risk Factor for Stroke. Psychosomatic Medicine 62.
- Kaczynski AT, Manske SR, Mannell RC, Grewal K. 2008. Smoking and physical activity: a systematic review. American Journal of Health Behavior.
- Van der Kooy K, van Hout H, Marwijk H, Marten H, Stehouwer C, Beekman A. 2007. Depression and the risk for cardiovascular diseases: Systematic review and meta analysis. International Journal of Geriatric Psychiatry 22: 613–626. doi: 10.1002/gps.1723
- Lindström M, Hanson BS, Östergren P-O. 2001. Socioeconomic differences in leisure-time physical activity: the role of social participation and social capital in shaping health related behaviour. Social Science & Medicine 52: 441–451. doi: https://doi.org/10.1016/S0277-9536(00)00153-2
- Lovejoy JC, Sainsbury A. 2009. Sex differences in obesity and the regulation of energy homeostasis: Etiology and pathophysiology. Obesity Reviews 10: 154–167. doi: 10.1111/j.1467-789X.2008.00529.x
- Maccioni L, Weber S, Elgizouli M, Stoehlker A-S, Geist I, Peter H-H, Vach W, Nieters A. 2018. Obesity and risk of respiratory tract infections: results of an infection-diary based cohort study. BMC Public Health 18: 271. doi: 10.1186/s12889-018-5172-8
- McDonald HI, Thomas SL, Nitsch D. 2014. Chronic kidney disease as a risk factor for acute community-acquired infections in high-income countries: a systematic review. BMJ Open 4: e004100. doi: 10.1136/bmjopen-2013-004100
- Meijers WC, de Boer RA. 2019. Common risk factors for heart failure and cancer. Cardiovascular research 115: 844–853. doi: 10.1093/cvr/cvz035

- Mitchell AJ, Chan M, Bhatti H, Halton M, Grassi L, Johansen C, Meader N. 2011. Prevalence of depression, anxiety, and adjustment disorder in oncological, haematological, and palliative-care settings: A meta-analysis of 94 interview-based studies. The Lancet Oncology 12: 160–174. doi: 10.1016/S1470-2045(11)70002-X
- Ng DM, Jeffery RW. 2003. Relationships between perceived stress and health behaviors in a sample of working adults. Health psychology : official journal of the Division of Health Psychology, American Psychological Association 22: 638–642. doi: 10.1037/0278-6133.22.6.638
- Novotny M, Klimova B, Valis M. 2018. PPI Long Term Use: Risk of Neurological Adverse Events? Frontiers in neurology 9: 1142. doi: 10.3389/fneur.2018.01142
- Paavola M, Vartiainen E, Haukkala A. 2004. Smoking, alcohol use, and physical activity: A 13-year longitudinal study ranging from adolescence into adulthood. Journal of Adolescent Health 35: 238–244. doi: https://doi.org/10.1016/j.jadohealth.2003.12.004
- Palmer SC, Vecchio M, Craig JC, Tonelli M, Johnson DW, Nicolucci A, Pellegrini F, Saglimbene V, Logroscino G, Hedayati SS, Strippoli GFM. 2013. Association between depression and death in people with CKD: a meta-analysis of cohort studies. American journal of kidney diseases : the official journal of the National Kidney Foundation 62: 493–505. doi: 10.1053/j.ajkd.2013.02.369
- Pampel FC. 2006. Global Patterns and Determinants of Sex Differences in Smoking. International Journal of Comparative Sociology 47: 466–487. doi: 10.1177/0020715206070267
- Pan XR, Yang WY, Li GW, Liu J. 1997. Prevalence of diabetes and its risk factors in China, 1994. National Diabetes Prevention and Control Cooperative Group. Diabetes care 20: 1664–1669. doi: 10.2337/diacare.20.11.1664
- Peleg AY, Weerarathna T, McCarthy JS, Davis TME. 2007. Common infections in diabetes: pathogenesis, management and relationship to glycaemic control. Diabetes/Metabolism Research and Reviews 23: 3–13. doi: https://doi.org/10.1002/dmrr.682
- Plieger T, Melchers M, Montag C, Meermann R, Reuter M. 2015. Life stress as potential risk factor for depression and burnout. Burnout Research 2: 19–24. doi: https://doi.org/10.1016/j.burn.2015.03.001
- Radloff L. 1975. Sex differences in depression - The effects of occupation and marital status. Sex Roles 1: 249–265. doi: 10.1007/BF00287373
- Ramirez JL, Drudi LM, Grenon SM. 2018. Review of biologic and behavioral risk factors linking depression and peripheral artery disease. Vascular Medicine (United Kingdom) 23: 478–488. doi: 10.1177/1358863X18773161
- Renn BN, Feliciano L, Segal DL. 2011. The bidirectional relationship of depression and diabetes: A systematic review. Clinical Psychology Review 31: 1239–1246. doi: https://doi.org/10.1016/j.cpr.2011.08.001
- Roberts RE, Deleger S, Strawbridge WJ, Kaplan GA. 2003. Prospective association between obesity and depression: Evidence from the Alameda County Study. International Journal of Obesity 27: 514–521. doi: 10.1038/sj.ijo.0802204
- Rundek T, Sacco RL. 2008. Risk factor management to prevent first stroke. Neurologic clinics 26: 1007–45, ix. doi: 10.1016/j.ncl.2008.09.001
- Schneider C, Jick SS, Bothner U, Meier CR. 2010. COPD and the Risk of Depression. Chest 137: 341–347. doi: https://doi.org/10.1378/chest.09-0614
- Sethi S. 2010. Infection as a comorbidity of COPD. European Respiratory Journal 35: 1209–1215. doi: 10.1183/09031936.00081409
- Stahl ST, Beach SR, Musa D, Schulz R. 2017. Living alone and depression: the modifying role of the perceived neighborhood environment. Aging & mental health 21: 1065–1071. doi: 10.1080/13607863.2016.1191060
- Stults-Kolehmainen MA, Sinha R. 2014. The Effects of Stress on Physical Activity and Exercise. Sports Medicine 44: 81–121. doi: 10.1007/s40279-013-0090-5
- Teychenne M, Ball K, Salmon J. 2008. Physical activity and likelihood of depression in adults: A review. Preventive Medicine 46: 397–411. doi: https://doi.org/10.1016/j.ypmed.2008.01.009
- Torres A, Peetermans WE, Viegi G, Blasi F. 2013. Risk factors for community-acquired pneumonia in adults in Europe: a literature review. Thorax 68: 1057–1065. doi: 10.1136/thoraxjnl-2013-204282
- Toskala E, Kennedy DW. 2015. Asthma risk factors. International forum of allergy & rhinology 5 Suppl 1: S11-6. doi: 10.1002/alr.21557
- Vankim NA, Nelson TF. 2013. Vigorous physical activity, mental health, perceived stress, and socializing among college students. American Journal of Health Promotion 28: 7–15. doi: 10.4278/ajhp.111101-QUAN-395

- Vijayan VK. 2013. Chronic obstructive pulmonary disease. The Indian journal of medical research 137: 251–269.
- Yusuf S, Hawken S, Ôunpuu S, Dans T, Avezum A, Lanas F, McQueen M, Budaj A, Pais P, Varigos J, Lisheng L. 2004. Effect of potentially modifiable risk factors associated with myocardial infarction in 52 countries (the INTERHEART study): case-control study. The Lancet 364: 937–952. doi: https://doi.org/10.1016/S0140-6736(04)17018-9
