# Supplementary material for: Major depressive disorders increase the susceptibility to self-reported infections in two German cohort studies
Source: Soc Psychiatry Psychiatr Epidemiol. 2022 Jul 5;58(2):277–86. doi: 10.1007/s00127-022-02328-5 (PMC9922209; doi:10.1007/s00127-022-02328-5)
Supplement: Supplementary file 4 — Supplementary file4 (PDF 152 KB) [file 127_2022_2328_MOESM4_ESM.pdf]

Article title
Major depressive disorders increase the susceptibility to self-reported infections in two German cohort studies

Journal name
Social Psychiatry and Psychiatric Epidemiology

Author names and affiliations
Henning Elpers[1], Henning Teismann, PhD[1], Jürgen Wellmann, PhD[1], Klaus Berger, MD[1], André Karch, MD[1], Nicole Rübsamen, PhD[1,*]

[1] Institute of Epidemiology and Social Medicine, University of Münster, Germany.
[*] Corresponding author:
Nicole Rübsamen | Institute of Epidemiology and Social Medicine | University of Münster | Albert-Schweitzer-Campus 1 | 48149 Münster | Germany
clinepi@uni-muenster.de

Online Resource 3: Results of the statistical tests of the conditional independencies implied by the directed acyclic graph

**Implied conditional independencies**	**Estimate**	**p-value**
Age ⫫ Sex	0	0.96
PPI ⫫ PhysicalActivity	0	0.96
COPD ⫫ HouseholdSize	0	0.93
COPD ⫫ Stress | Smoking	-0.01	0.86
Asthma ⫫ PhysicalActivity | BMI, MDD, Smoking, Stress	-0.01	0.86
BMI ⫫ Smoking | PhysicalActivity, SES, Sex	-0.01	0.81
Diabetes ⫫ HouseholdSize	-0.01	0.81
Alcohol ⫫ Stress	0.01	0.76
Age ⫫ Smoking	-0.01	0.75
Alcohol ⫫ PhysicalActivity | SES, Smoking, Stress	0.01	0.73
PPI ⫫ Sex	-0.01	0.67
Alcohol ⫫ HouseholdSize	-0.01	0.65
COPD ⫫ PhysicalActivity | Smoking	-0.02	0.64
Asthma ⫫ HouseholdSize | BMI, MDD, Smoking, Stress	0.02	0.63
HF ⫫ HouseholdSize | Age, Alcohol, BMI, CKD, Diabetes, MDD, PhysicalActivity, SES, Sex, Smoking, Stress, Stroke	0.02	0.55
BMI ⫫ Stress | PhysicalActivity, SES, Sex	-0.02	0.54
BMI ⫫ HouseholdSize	-0.02	0.54
Asthma ⫫ COPD | BMI, MDD, Smoking, Stress	-0.02	0.53
Sex ⫫ Stress	-0.02	0.51
Asthma ⫫ Sex | BMI, MDD, Smoking, Stress	0.02	0.47
Alcohol ⫫ Asthma | BMI, MDD, Smoking, Stress	-0.02	0.47
COPD ⫫ Sex | Smoking	0.03	0.4
PPI ⫫ Smoking	0.03	0.38
Age ⫫ COPD	0.03	0.32

Implied conditional independencies	Estimate	p-value
Asthma ⫫ Diabetes | BMI, MDD, Smoking, Stress	0.03	0.29
Age ⫫ PPI	0.04	0.28
SES ⫫ Stress	-0.04	0.2
Asthma ⫫ Stroke | BMI, MDD, Smoking, Stress	-0.04	0.2
CKD ⫫ Sex | Alcohol, BMI, PhysicalActivity, SES, Smoking, Stress	-0.04	0.19
Alcohol ⫫ COPD | Smoking	-0.04	0.18
Age ⫫ BMI | PhysicalActivity, SES, Smoking, Stress	-0.04	0.18
Age ⫫ Asthma | BMI, MDD, Smoking, Stress	0.05	0.16
Age ⫫ BMI | PhysicalActivity, SES, Sex	-0.05	0.14
Asthma ⫫ SES | BMI, MDD, Smoking, Stress	-0.05	0.14
CKD ⫫ HouseholdSize	-0.05	0.14
Asthma ⫫ CKD | BMI, MDD, Smoking, Stress	0.05	0.11
Alcohol ⫫ PPI	-0.05	0.1
CKD ⫫ COPD | Smoking	0.06	0.089
COPD ⫫ Diabetes | Smoking	0.06	0.083
HouseholdSize ⫫ SES	0.06	0.07
HouseholdSize ⫫ Stress	-0.06	0.068
Asthma ⫫ PPI | BMI, MDD, Smoking, Stress	0.06	0.061
Age ⫫ Alcohol	0.06	0.049
COPD ⫫ SES | Smoking	-0.07	0.037
Diabetes ⫫ Sex | Alcohol, BMI, PhysicalActivity, SES, Smoking, Stress	-0.07	0.037
COPD ⫫ HF | Age, Alcohol, BMI, CKD, Diabetes, MDD, PhysicalActivity, SES, Sex, Smoking, Stress, Stroke	0.07	0.029
BMI ⫫ COPD | PhysicalActivity, SES, Sex	0.08	0.021
CKD ⫫ PPI	0.08	0.013
HouseholdSize ⫫ Smoking	-0.08	0.011
Age ⫫ SES	-0.08	0.011
Alcohol ⫫ SES	0.08	0.01
Asthma ⫫ HF | BMI, MDD, Smoking, Stress	0.09	0.0096
BMI ⫫ COPD | Smoking	0.09	0.0095
COPD ⫫ PPI	0.09	0.0093
Alcohol ⫫ BMI | PhysicalActivity, SES, Sex	-0.09	0.0083
PPI ⫫ Stress	-0.09	0.0075
Alcohol ⫫ BMI | SES, Sex, Smoking, Stress	-0.09	0.0068
HF ⫫ PPI | Age, Alcohol, BMI, CKD, Diabetes, MDD, PhysicalActivity, SES, Sex, Smoking, Stress, Stroke	0.09	0.0061
HouseholdSize ⫫ PPI	-0.1	0.0029
HouseholdSize ⫫ PhysicalActivity	0.12	0.00032
HouseholdSize ⫫ Sex	-0.12	0.00031
PhysicalActivity ⫫ Sex | SES, Smoking, Stress	0.12	0.00028
SES ⫫ Sex	-0.13	<0.0001
PPI ⫫ SES	-0.15	<0.0001
Age ⫫ Stress	0.16	<0.0001
BMI ⫫ PPI	0.18	<0.0001

**Implied conditional independencies**	**Estimate**	**p-value**
Diabetes ⫫ PPI	0.19	<0.0001
Age ⫫ HouseholdSize	-0.25	<0.0001

Only conditional independencies bewteen measured variables were tested.
