# Supplementary material for: Major depressive disorders increase the susceptibility to self-reported infections in two German cohort studies
Source: Soc Psychiatry Psychiatr Epidemiol. 2022 Jul 5;58(2):277–86. doi: 10.1007/s00127-022-02328-5 (PMC9922209; doi:10.1007/s00127-022-02328-5)
Supplement: Supplementary file 5 — Supplementary file5 (PDF 640 KB) [file 127_2022_2328_MOESM5_ESM.pdf]

Article title

Major depressive disorders increase the susceptibility to self-reported infections in two German cohort studies

Journal name

Social Psychiatry and Psychiatric Epidemiology

Author names and affiliations

Henning Elpers[1], Henning Teismann, PhD[1], Jürgen Wellmann, PhD[1], Klaus Berger, MD[1], André Karch, MD[1], Nicole Rübsamen, PhD[1,*]

[1] Institute of Epidemiology and Social Medicine, University of Münster, Germany.
[*] Corresponding author:
Nicole Rübsamen | Institute of Epidemiology and Social Medicine | University of Münster | Albert-Schweitzer-Campus 1 | 48149 Münster | Germany
clinepi@uni-muenster.de

Online Resource 4: p value functions

P value function of the effect of MDD on URTI in the BiDirect (n=925) and HaBIDS (n=1,007) cohort study

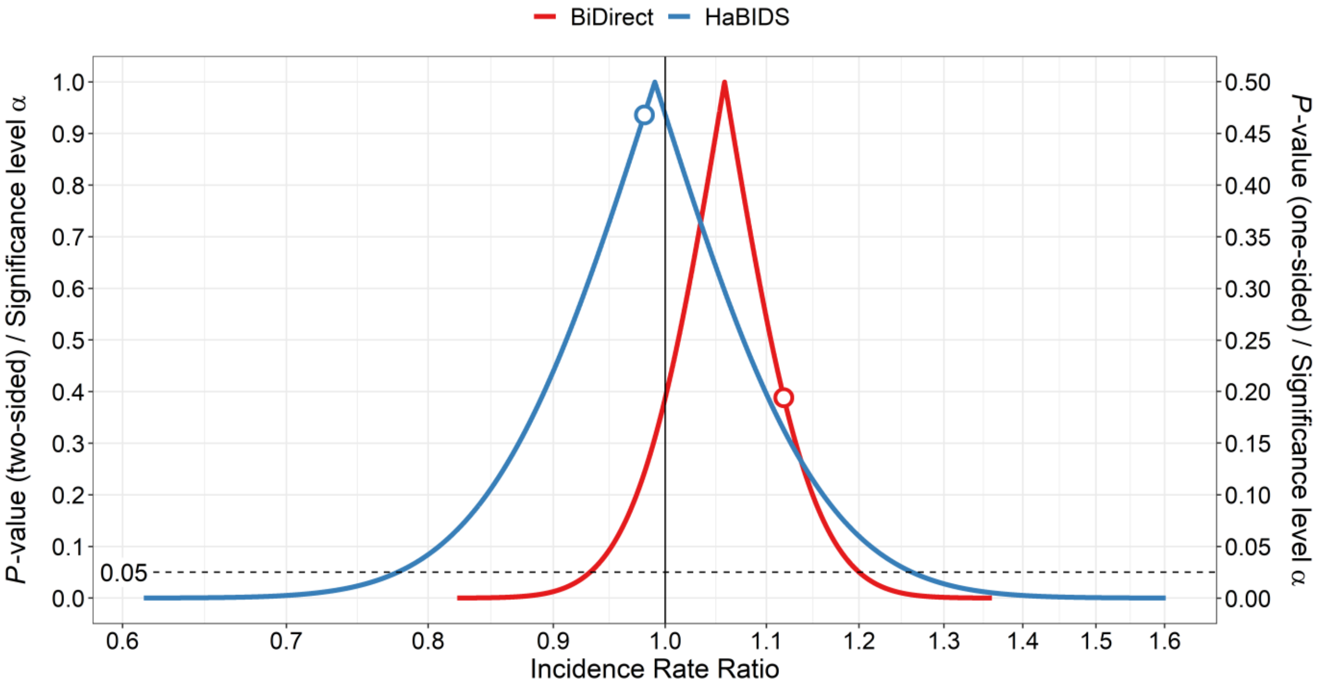

The circle represents the counternull incidence rate ratio.

P value function of the effect of MDD on LRTI in the BiDirect (n=925) and HaBIDS (n=1,007) cohort study

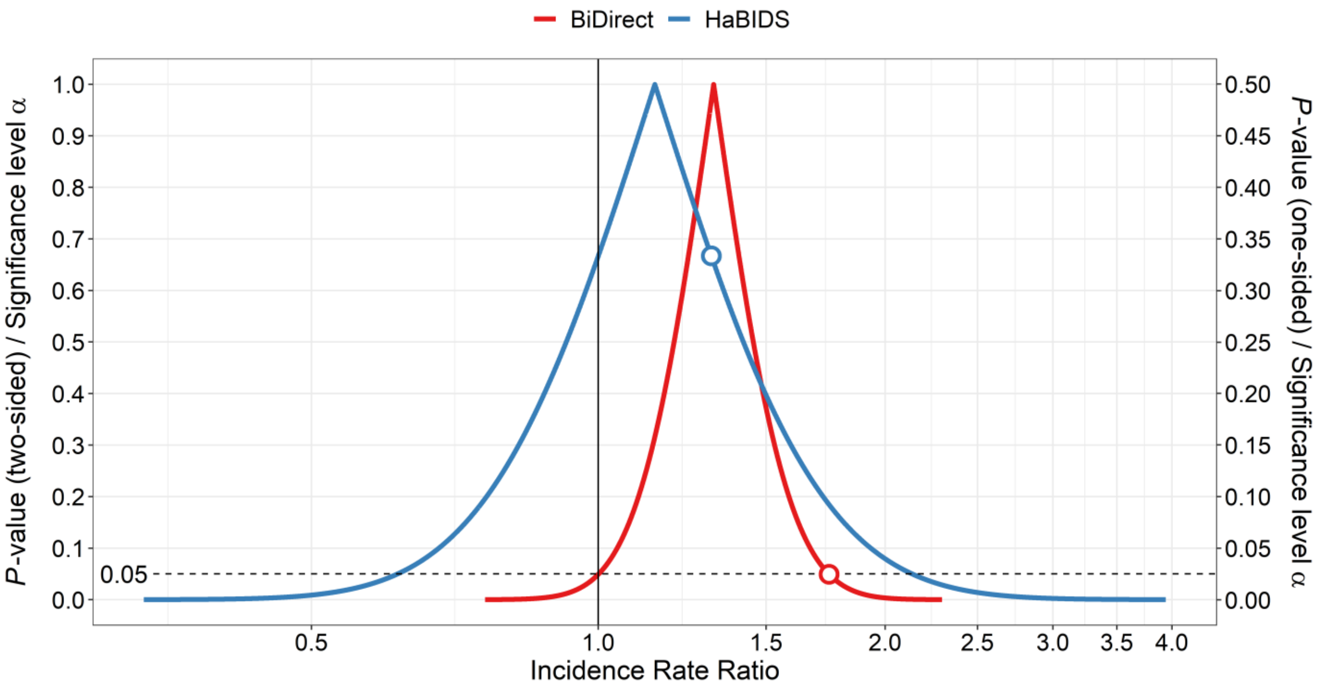

The circle represents the counternull incidence rate ratio.

P value function of the effect of MDD on any RTI in the BiDirect (n=925) and HaBIDS (n=1,007) cohort study

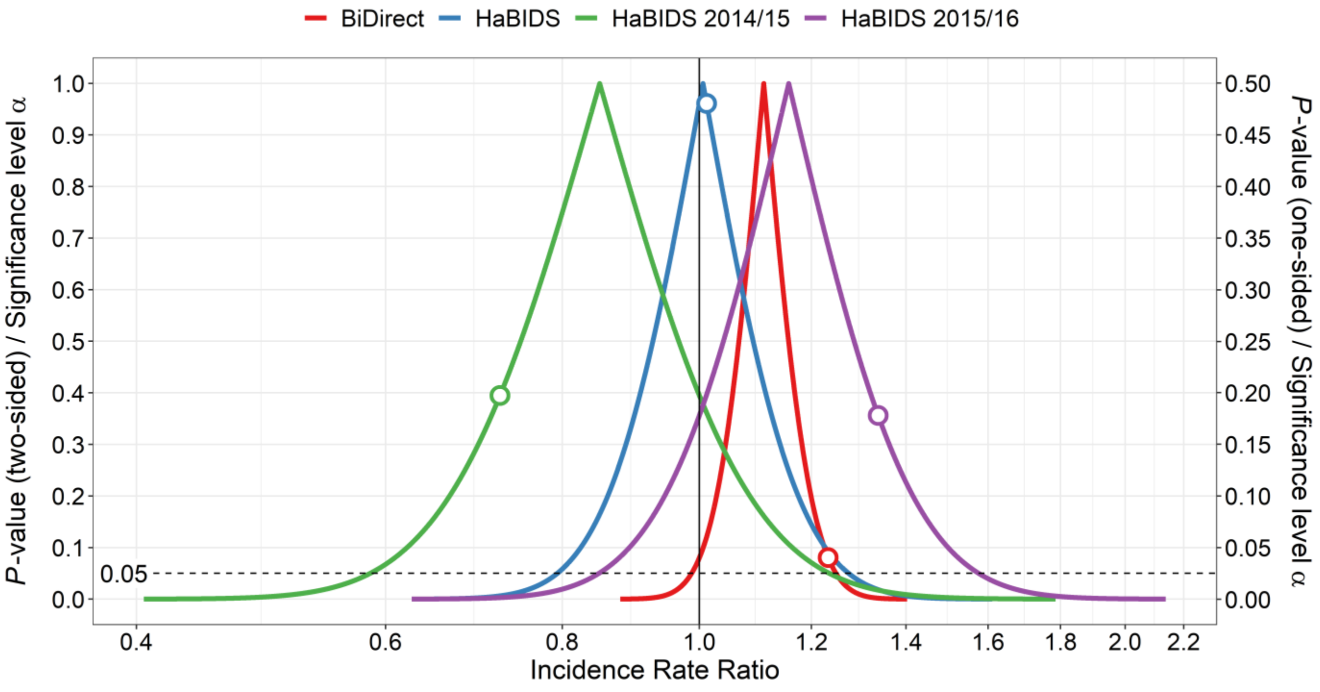

The circle represents the counternull incidence rate ratio.

P value function of the effect of MDD on Cystitis in the BiDirect (n=925) and HaBIDS (n=1,007) cohort study

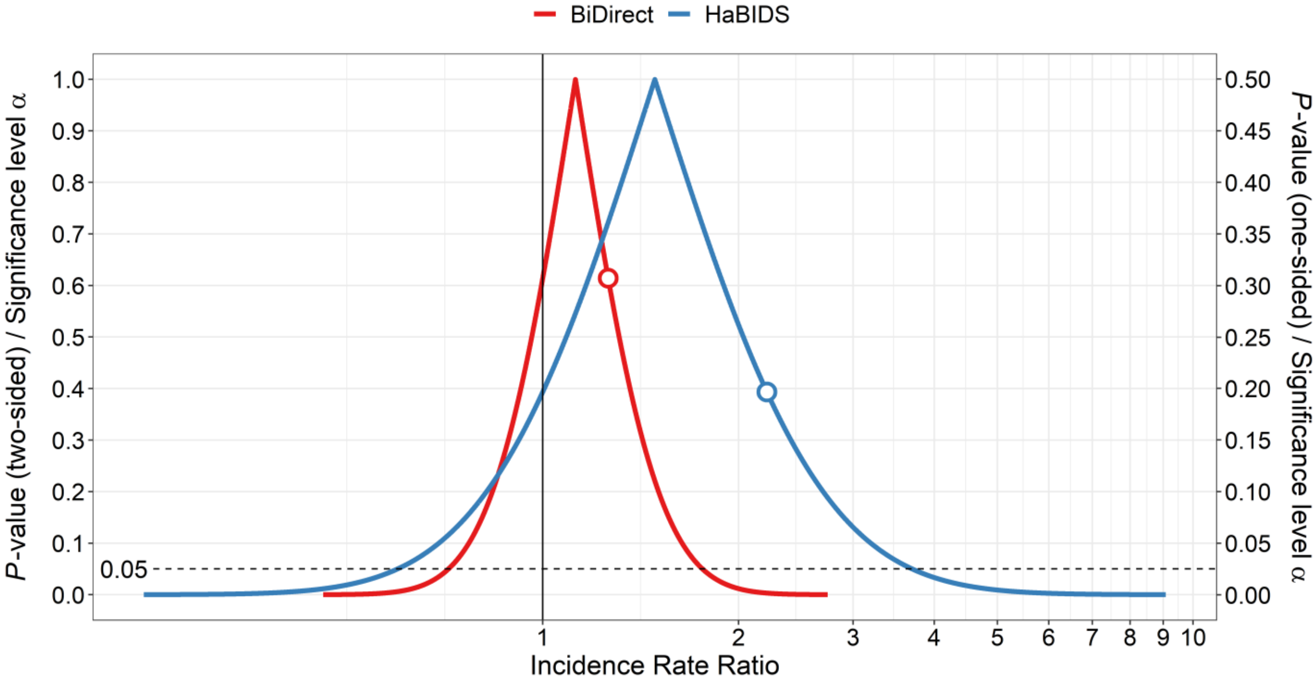

The circle represents the counternull incidence rate ratio.

P value function of the effect of MDD on fever in the BiDirect (n=925) and HaBIDS (n=1,007) cohort study

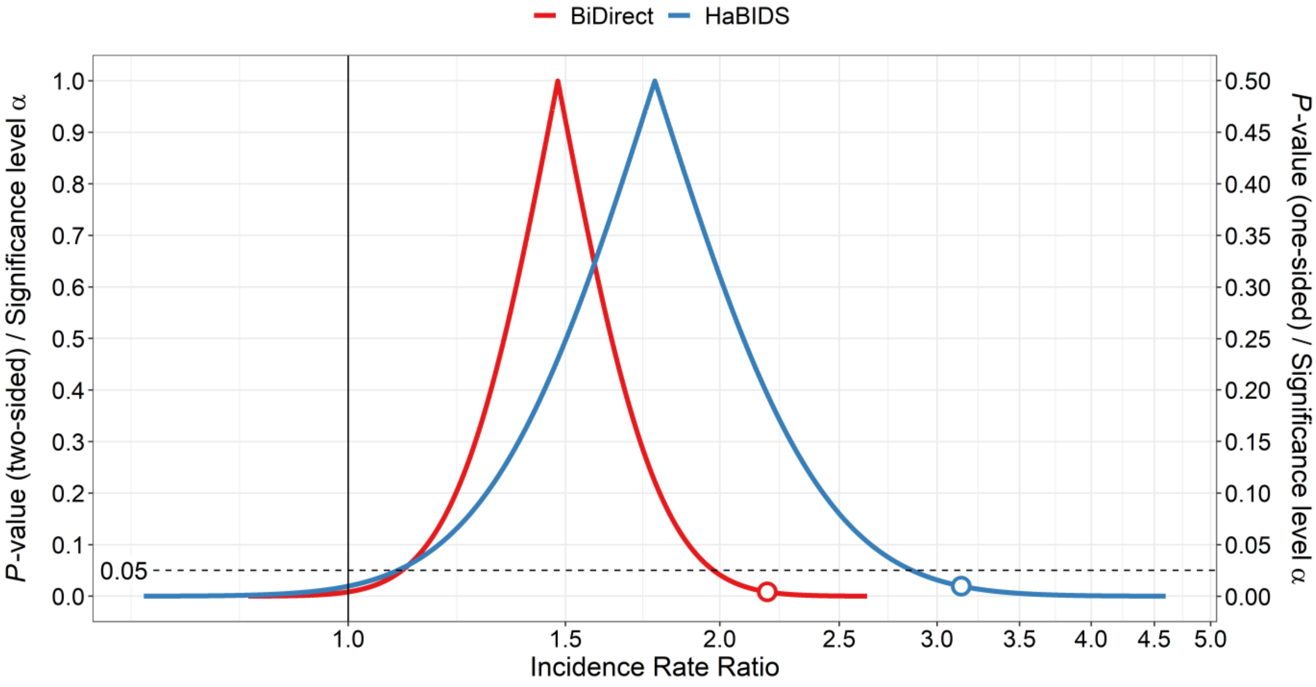

The circle represents the counternull incidence rate ratio.
